# 

**Published:** 1916-08-12

**Authors:** 


### The College of Nursing, Limited.

### OFFICIAL NOTICE: CERTIFICATES OF TRAINING.

The following official statement is being issued by the Council of the College :—

The Council of the College of Nursing, Ltd., in order to meet the convenience of matrons, sisters, and nurses on active service, has waived the obligation of candidates for registration to forward, with their application forms, copies of the certificates they hold from their nurse-training schools. These certificates, however, will be required later, that they may be filed.

It is felt that this will be a great convenience to nurses abroad, also those serving in military hospitals at home will be glad to be relieved of the difficulty of procuring their certificates, which in many cases are stored with other personal possessions.

The Council would like the nurses who have sent in their application forms, and who may not yet have received any further communication, to know that the delay has been in consequence of some necessary alterations in the articles of association, that the formalities of registration are being proceeded with, and as soon as possible will be completed.

### Brave and Gallant Medical Officers.

Further brilliant records of gallantry and devotion to duty on the part of officers in the Medical Services engaged with the Forces are contained in the list, published at the end of last week, of appointments to the Distinguished Service Order which His Majesty has been graciously pleased to approve and in the list of those on whom the Military Cross has been conferred. The D.S.O. has been gained by four such officers—to wit, Temporary Captain Walter Dawson, M.B., R.A.M.C.; Captain Peter Fleming Gow, M.B., Indian Medical Service; Captain William Archibald Miller, M.B., R.A.M.C.; and Temporary Lieutenant Lionel Matthew Rowlette, R.A.M.C. The deeds performed by these men are in accord with the highest traditions of the Service—searching, under heavy fire, for wounded men, and other deeds equally daring. The following twenty officers are the recipients of the Military Cross: Temporary Captain Norman Black, M.B., R.A.M.C.; Temporary Captain C. M. Brophy, R.A.M.C.; Captain J. A. Cullum, Canadian A.M.C.; Captain H. E. Cumming, Canadian A.M.C.; Captain H. F. L. Hugo, M.B., R.A.M.C.; Captain A. E. Ironside, R.A.M.C.; Temporary Captain F. P. Jocelyne, R.A.M.C.; Captain N. Lockhart Joynt, M.B., R.A.M.C.; Captain R. L. Lloyd, South African Medical Corps; Temporary Captain I. M. Mackenzie, M.B., R.A.M.C.; Captain W. H. Riddell, M.B., I.M.S.; Captain R. A. Stewart, M.B., R.A.M.C.; Captain G. W. Treleaven, Canadian A.M.C.; Temporary Lieutenant J. R. Irwin, M.B., R.A.M.C.; Temporary Lieutenant C. C. Okell, R.A.M.C.; Temporary Lieutenant P. M. Turnbull, M.B., R.A.M.C.; Temporary Captain H. A. Pallant, R.A.M.C.; Temporary Lieutenant F. R. Hassard, M.B., R.A.M.C.; Temporary Lieutenant V. F. Stock, M.B., R.A.M.C.; Temporary Lieutenant G. B. Wiswell, M.B., R.A.M.C. All these men have performed deeds of cool and cheerful bravery, disregarding their own safety and comfort so that the lives of others may be saved.

Newington House, Edinburgh, has been officially recognised as a hospital and training school for wounded soldiers, and the aim is to make it a sort of Scottish St. Dunstan's. It is under the control of the Edinburgh Royal Blind Asylum.

### Matrons' and Sisters' Appointments.

Beckett Hospital, Barnsley.—Miss Annie Louisa Burkhill has been appointed matron. She was trained at Manchester Royal Infirmary, and has since been assistant matron at Manchester Infectious Diseases Hospital and matron of the Beverley Dispensary, E. Yorks.

Eastby Sanatorium, near Skipton.—Miss S. E. Hutton has been appointed matron. She was trained at the Children's Hospital and the Royal Infirmary, Edinburgh; did district nursing at Edinburgh and Hull; was sister of Hull Sanatorium; matron of Howden Isolation Hospital; matron of Kirkburton Isolation Hospital; matron of the Columbia Coast Mission Hospital, Van Anda, Canada; assistant superintendent of the Los Angeles Children's Hospital, California; Red Cross sister at Leeds and Weymouth.

Oldham Royal Infirmary.—Miss Mary Mackintosh has been appointed matron. She was trained at the Royal Southern Hospital, Liverpool, where she has since been assistant matron.

Cricklade and Wootton Bassett Isolation Hospital.—Miss Mary E. Armitt has been appointed nurse-matron. She was trained at the Monsall Fever Hospital, Manchester, and Leeds Maternity Hospital, Leeds. She has since been assistant nurse at Crosland Moor Infirmary, Huddersfield; staff nurse and charge nurse at Ruchill Fever Hospital, Glasgow; district health visitor for Normanton (Yorks); charge nurse of the Meltham Isolation Hospital, near Huddersfield; night charge nurse at Rothwell Isolation Hospital, Leeds. She has also done private nursing, and holds the certificate of the Central Midwives Board.

Darlington Hospital.—Miss Elsie Collins has been appointed sister and Miss Richardson night sister. Miss Collins was trained at the Northampton General Hospital, where she has since been sister. After serving with Queen Alexandra's Imperial Military Nursing Service Reserve she returned to Northampton in charge of the theatre and a soldiers' pavilion Miss Richardson was trained at the Chelmsford Hospital, and has since been staff nurse at the Stockton and Thornaby Hospital, night charge nurse at the Manchester Victoria Hospital, charge nurse at the Walker Accident Hospital, sister at Cumberland Infirmary, and night sister at Merthyr General Hospital.

Royal Sussex County Hospital, Brighton.—Miss Annie M. Latham has been appointed acting sister-housekeeper. She was trained at Leeds General Infirmary, and has since been theatre and night sister at St. Bartholomew's Hospital, Rochester.

Skipton Isolation Hospital.—Miss Lilian Davies has been appointed ward sister. She was trained at the Ladywell Sanatorium and the Royal Infirmary, Blackburn. She has since been charge nurse at the former, and ward sister at the latter.

Stirnesdale Sanatorium, Oldham.—Miss Eleanor May Harding has been appointed sister-in-charge. She was trained at Marylebone Infirmary, and has since been assistant matron at High Carley Sanatorium, Ulverston.

**NOTE.—Particulars of Appointments for insertion in this column will be welcomed.**

### Over Fifteen Hundred Pounds for the Bradford Royal Infirmary.

The Treasurer of the Bradford Royal Infirmary has received £450 bequeathed by the late Sir Theo. Peel, Bart., and a cheque for £1,000 from the Executive Committee of the Bradford Hospital and Convalescent Fund, being five-eighths of a sum which they have allocated among the medical charities of Bradford, while the executors of the late Mr. John Conchar, of Bradford, have intimated that a sum of £100 has been bequeathed to the Infirmary.

### Passing Events and Topics.

### Scholarships at University College of London.

The following awards have been made: Bucknill Scholarship (Faculty of Medical Sciences) and First Medical Entrance Exhibition: Cadman, Maurice Danks, of the Grammar School, Newport (Salop), and Harris, Henry Albert, of University College, Cardiff (divided equally). Second Medical Entrance Exhibition: Goldwater, Harry Gerald, of Rhodes University College, Grahamstown, South Africa.

### The National Institute for the Blind.

The success of the National Institute is no doubt partly due to its policy of co-ordination in the blind charity world. The Institute (formerly known as the British and Foreign Blind Association) now has under its wing four other charities, which are managed by separate committees as branches and subsidised by the parent institution. The total income of the Institute last year was £34,997, and the expenditure £32,776. There is a "Treasurer's Fund," the reason for which is not apparent, which had an income of £25,334, of which sum £6,000 was allocated to the General Fund.

### A Record Balance-Sheet.

It was announced by the hon. treasurer, Mr. G. F. Grant, at the annual meeting of the Victoria Hospital for Sick Children, Hull, that £600 had been received from the Hull branch of the Women Workers' League to endow a cot in memory of Dr. Mary Murdoch, the senior honorary physician of the hospital, and that a further sum of £352 would be forthcoming from the same source. Mr. T. R. Ferens, M.P., who presided, mentioned that the institution had never had a more favourable balance-sheet than that for 1915, the adverse balance of £1,288 having been converted into a credit balance of £287.

### Saving the Babies.

Following a recommendation from the Local Government Board, Clydebank Town Council is co-operating with the School Board in the establishment of an Infant Welfare Centre. The infant death-rate in the Clyde area has been mounting up higher and higher, and the experience of Motherwell, where the mortality was halved in one year after the adoption of a Child Welfare scheme, speaks volumes for what it is possible to accomplish. An Infant Welfare Centre which owes its inception largely to the enthusiasm of the Mayoress (Mrs. H. C. Jones), who has equipped it at her own expense, has been opened at Nuneaton.

### Tuberculosis Institutions in Canada.

Tuberculosis sanatoriums in Canada now have accommodation for 1,800 beds, or one bed for every 4,407 of the population. All of these, says the official organ of the American Medical Association, with the exception of one in Nova Scotia, have been the result of private philanthropy, assisted later on by municipal and provincial Governments. The Province of Ontario is best supplied with sanatoriums, there being one bed to 2,403 of the population. Manitoba comes second in the matter of provision for the tuberculous. As a result, in Ontario there has been a very marked decrease in the deaths from tuberculosis; Manitoba comes second in the decrease of the death-rate.

Rates of Subscription: Twelve months, 6/6; Six months, 4/-; Three months, 2/-, post free to any address in the United Kingdom. Abroad, Twelve months, 8/8; Six months, 5/-; Three months, 2/6, post free. The Hospital "Index" to Volume LIX., October 1915 to March 1916, is now ready, price 1½d. per copy, post free. Address The Manager, "The Hospital," The Hospital Building, 28/29 Southampton Street, Strand, London, W.C.

